# From Variation of Influenza Viral Proteins to Vaccine Development

**DOI:** 10.3390/ijms18071554

**Published:** 2017-07-18

**Authors:** Wandi Zhu, Chao Wang, Bao-Zhong Wang

**Affiliations:** Center for Inflammation, Immunity & Infection, Georgia State University Institute for Biomedical Sciences, Atlanta, GA 30303, USA; wzhu3@gsu.edu (W.Z.); cwang45@gsu.edu (C.W.)

**Keywords:** influenza virus, antigenic drift and shift, universal influenza vaccine

## Abstract

Recurrent influenza epidemics and occasional pandemics are one of the most important global public health concerns and are major causes of human morbidity and mortality. Influenza viruses can evolve through antigen drift and shift to overcome the barriers of human immunity, leading to host adaption and transmission. Mechanisms underlying this viral evolution are gradually being elucidated. Vaccination is an effective method for the prevention of influenza virus infection. However, the emergence of novel viruses, including the 2009 pandemic influenza A (H1N1), the avian influenza A virus (H7N9), and the highly pathogenic avian influenza A virus (HPAI H5N1), that have infected human populations frequently in recent years reveals the tremendous challenges to the current influenza vaccine strategy. A better vaccine that provides protection against a wide spectrum of various influenza viruses and long-lasting immunity is urgently required. Here, we review the evolutionary changes of several important influenza proteins and the influence of these changes on viral antigenicity, host adaption, and viral pathogenicity. Furthermore, we discuss the development of a potent universal influenza vaccine based on this knowledge.

## 1. Introduction

Influenza viruses are fragmented, negative-strand RNA viruses belonging to the family Orthomyxoviridae. Influenza viruses A, B, C, and D compose the four influenza virus genera in the family [1,2]. Of them, influenza A viruses can infect a wide variety of animal species, including birds, pigs, humans, horses, dogs, and other hosts. Meanwhile, influenza B viruses are only found in humans, although they possess a similar structure to influenza A viruses. Compared with influenza A and B viruses which have eight discrete gene segments, influenza C and D viruses contain only seven genomic segments [3]. Influenza C viruses predominately infect humans, although infections in pigs, dogs, horses, and cattle have been observed. Influenza D viruses, recently isolated from swine and cattle, has also been identified in sheep and goats [4,5]. Influenza A viruses have been demonstrated to be the most severe risk for zoonotic infection and human influenza pandemics, and will be the focus of this review.

Both influenza A and B viruses cause seasonal epidemics each year, with 3 to 5 million infections and 250,000 to 500,000 deaths worldwide [6]. Over 200,000 hospitalizations and 30,000 to 50,000 deaths are attributed to seasonal influenza infection in the United States annually [7,8]. Some groups, including the elderly, infants, children under 5 years old, pregnant women, and people with chronic diseases, are at high risk of influenza infection with increased mortality rates [9,10,11,12,13]. In addition to annual seasonal outbreaks, influenza pandemics also occur occasionally. In the past 200 years, there have been five pandemics: the 1918 H1N1 Spanish flu pandemic, the 1957 H2N2 Asian pandemic, the 1968 H3N2 Hong Kong pandemic, the 1977 H1N1 pandemic, and the 2009 H1N1 pandemic [14]. The H5N1 highly pathogenic avian influenza (HPAI), which derived from the 1996 Chinese epizootic virus, evolved rapidly and might become an agent for a future pandemic. From 2003 to 2017, the World Health Organization (WHO) reported that 453 cases of deaths happened in a total of 858 confirmed human cases of H5N1 infection, which indicated that H5N1 HPAI leads to more than 50% mortality in humans [15]. In addition, some avian H7 and H9 subtypes, such as H9N2 LPAI (low pathogenic avian influenza), H7N7 HPAI, and H7N3 HPAI, have been reported to cause human infections [16,17,18]. To date, the avian influenza A H7N9, which was first reported in China in 2013, has induced five seasonal outbreaks and increased human infections and deaths [19].

Influenza viruses harbor RNA-dependent RNA polymerase that lack proofreading capability and tend to make errors during replication. This results in high mutation rates that aid in the evasion of host-established immunity. Moreover, because the influenza virus genome consists of discrete RNA segments, genetic re-assortment can occur easily when two different influenza viruses co-infect the same host [20]. Both antigenic drift and shift are very important strategies for the influenza viral evolution, zoonotic infection, and transmission. Antigenic drift and shift present major challenges for current vaccines and antiviral drugs. This review will discuss the genetic variation of influenza virus and its effect on the development of a universal influenza vaccine. Table 1 summarizes the mutation sites which are critical for the function of influenza viral proteins, viral infection, host adaption, and transmission, and lists their application in vaccine development.

## 2. Relationship between Influenza Virus Proteins and Vaccine Development

### 2.1. Hemagglutinin

**Figure 1:**
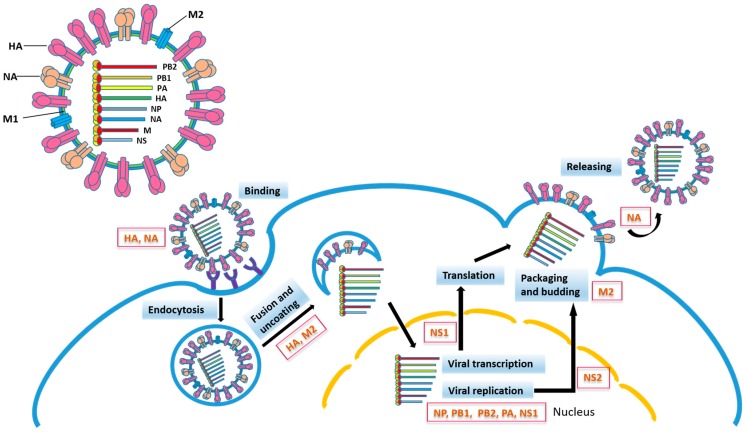
Influenza virus particle and life cycle. Influenza viral proteins participating in different steps of virus infection are indicated. HA, hemagglutinin; NA, neuraminidase; M1, matrix 1 protein; M2, matrix 2 protein; NP, nucleoprotein; PB2, polymerase basic protein 2; PB1, polymerase basic protein 1; PA, polymerase acidic protein; NS1, nonstructural protein 1; NS2, nuclear export protein.

The influenza hemagglutinin (HA) is a type I integral membrane protein. Its ectodomain consists of the outward globular HA1 domain and the HA2 stem domain, through which the ectodomain is associated with the membrane-spanning region which anchors the whole protein to the virus membrane. Based on the variability of HA, influenza A viruses are classified into 18 HA (H1-H18) subtypes [21]. The influenza virus life cycle is initiated by the recognition of sialic acid (SA) of the host cell glycoprotein by HA, followed by endocytosis. Fusion of the viral and endosomal membrane requires an acidic pH-triggered HA conformational change (Figure 1).

HA is the major antigen of influenza viruses and the most important target of host humoral immune response. Most antibodies produced in the host are against the immunodominant head domain. However, during the viral infection the most influential amino acid changes occur near the top of head domain—the receptor-binding sites which could influence the receptor binding affinity, specificity, and transmission [22]. This results in only a small number of the antibodies against the hemagglutinin globular head domain showing broad binding capabilities [23,24]. Despite the high divergence within the head domain, the stalk domain retains sequence conservation. The induction of broader HA stalk-specific antibodies is an important approach for the development of universal influenza virus vaccines [25]. It has been demonstrated that some stalk-reactive antibodies have broad binding activity to either or both group 1 and group 2 hemagglutinin, and even influenza B hemagglutinin [26,27,28,29,30]. Studies on the mechanism underlying the protective efficacy of stalk-reactive antibodies reveal that these antibodies can block membrane fusion of the virus and endosome by blocking the cleavage between HA1 and HA2 subunits, inhibiting viral budding through interaction with hemagglutinin on the cell surface, or antibody-dependent cell mediated cytotoxicity (ADCC) [31,32,33,34]. Chimeric hemagglutinins with different head domains have been used to induce high titers of stalk-specific antibodies by a sequential immunization strategy [25,35,36]. Recently, some strategies focusing on the reconstruction of HA to expose the HA stalk portion without interference of its native-like structures to trigger neutralizing antibodies have been reported, such as an HA-mini stem mimicking the HA prefusion conformation [37], a stitched HA stem only containing conserved fragments [38], and an engineered H3 subtype HA directly displaying H1 subtype HA stalk peptides on the head domain [39]. Furthermore, nanoparticles based on conserved HA stalk epitopes in the context of a heterologous prime-boost regimen is a promising method for the induction of broadly protective efficacy and long-lasting immune response [40].

Amino acid residue substitutions at glycosylation sites of HA molecules may also influence receptor binding, antigenic changes, and cluster transition [41]. For examples, the loss of glycosylation at 158–160 was critical for H5N1 influenza viruses to bind human receptor and to transmit among a mammalian host [42]. Lacking glycosylation at 158 N was the prerequisite for H5N1 binding to the α2,6-linked receptor [43]. Although many publications have demonstrated that glycosylation of hemagglutinin influences the virus virulence [44,45,46], whether the alteration of virus virulence caused by glycosylation modulating receptor binding ability needs to be further verified. A recent study showed that glycosylation changes in the H3N2 head domain altered the receptor binding ability of the virus but did not affect the virulence [47]. Modifying the glycosylation sites in the hemagglutinin head domain could be another effective method for regulating the immunogenicity of HA and presents a new idea for vaccine development. This was supported by a study showing that immunization with additional glycosylation sites in recombinant HA induced higher HA stalk-specific antibodies and better protection against a virus challenge [48]. A recent study demonstrated that an inactivated de-glycosylated influenza virus vaccine could induce cross-reactive neutralizing antibody responses and provided better cross-protection [43]. The glycosylation in the stalk region was relatively conserved and important for the folding of HA, viral intracellular transportation, and membrane fusion [49,50,51].

Mutations in the proteolytic cleavage site of HA influenced the pathogenicity of influenza virus as well. Low pathogenic influenza H5 and H7 viruses evolved into high pathogenic influenza viruses by acquiring polybasic amino acid insertion mutations at the HA cleavage sites. These mutations enhanced HA cleavage efficacy and were found to increase the virulence of H5N1 [52,53]. The generation of live attenuated vaccine strains and redesigned HA based on the cleavage site are two approaches to explore new candidates for influenza vaccines [54,55]. Not all mutations are beneficial for viral replication, but influenza viruses can find ways to overcome these obstacles. For example, poorly replicated clinical H3N2 strains can propagate in vitro by rapidly acquiring HA or NA mutations, which increased virus binding ability [56]. The replication of a virus containing an attenuating mutation in the HA receptor binding site can be modulated or compensated by other mutations in HA or NA that increase receptor binding avidity [57,58]. Thus, safety issues should be carefully considered and understood before applying a new live attenuated vaccine strain.

### 2.2. Neuraminidase

Influenza neuraminidase (NA) is a type II integral membrane protein possessing sialidase enzymatic activity that is required for the cleavage of SA from host cells and viral glycoproteins. The NA glycoprotein is a homotetramer composed of four identical subunits. Each subunit is made up of a cytoplasmic domain, a transmembrane domain, a stalk domain, and a globular head domain [59]. During virus replication, NA plays important roles in the release of new progenies and the prevention of their aggregation. In addition, NA has been shown to play a role in virus penetration through the mucus barrier of the respiratory tract by cleaving sialylated decoys [60,61,62].

Compared with the immunogenicity of hemagglutinin, neuraminidase is immunosubdominant and has lower antigenic drift rates, which introduces the possibility that the induction of a neuraminidase-based immune response could be a valuable approach for the development of universal influenza vaccines. Recent studies have shown that elicited NA immunity can provide protection against lethal homologous influenza virus infection, but limited cross-protection against heterologous virus infection [63,64,65]. In addition, more and more studies have indicated that NA has equal immunogenicity with HA and that the immunodominance of HA might be due to its high distribution on the viral surface [64,66,67]. The effective protective function of NA-induced humoral immunity has been gradually recognized [64,65,68,69,70]. Based on the methods used to promote high levels of HA stalk-specific antibodies, similar approaches could be employed to induce strong humoral responses against NA, such as vaccination with a chimeric virus with genetically engineered HA and NA, sequential immunization with identical NA but less immunogenic HA, application of NA-based nanoparticles, or the direct supplementation of NA vaccine into the current seasonal influenza vaccines.

Unlike the conservation of the HA stalk domain, the NA stalk domain is hypervariable. Several studies have demonstrated that the discrepancy of the NA stalk domain, including sequence deletion and glycosylation, could influence viral pathogenicity and transmission [71,72,73,74]. One study has shown that a conserved amino acid in the stalk region was critical for stabilizing the tetrameric structure and enzymatic activity of NA [75]. The enzymatic active site is in the NA head domain and is usually conserved among majority subtypes. The conserved epitopes in the enzymatic site can be a potential candidate for a universal influenza vaccine. For instance, an eight-amino acid sequence in the enzymatic active site was identified as universally conserved among all the influenza viruses and played important roles in viral replication [76]. Some mutations may occur on the surface loops surrounding the enzyme active site, which is also the target of the antiviral drugs oseltamivir and zanamivir against NA. Thus, these mutations in the active site could cause drug resistance. Monoclonal antibodies against the conserved sequence have shown broad inhibition against all influenza A NA, influenza B NA, as well as drug resistant strains [76,77].

### 2.3. Mutations in Other Influenza Virus Proteins

The influenza M2 ion channel protein is the third transmembrane protein consisting of an N-terminal ectodomain, a transmembrane domain, and a C-terminal intracellular domain [78]. The sequence of M2 protein is extremely conserved. The introduction of mutations could disrupt M2 function during infections [79]. The ectodomain of M2 (M2e) is a very promising candidate for the development of a universal influenza vaccine because of its high conservation. Many M2e-based vaccines have been constructed and tested in animal models for protective efficacy against different influenza virus infections, such as tetrameric M2e, M2e VLP (virus-like particle) vaccines, adjuvant conjugated M2e vaccines, and recombinant M2e protein containing M2e sequences from different species [80]. In addition, M2 is the target of the antiviral drug adamantanes which inhibits viral replication through directly binding to the pore of the ion channel [81]. However, in the past several decades, over 95% of drug resistant strains occurred due to the three mutations—V27A, S31N, and L26F in the pore-lining residues located in the transmembrane helix of M2, in which the notable S31N is the prevalent drug resistant mutation [82,83]. The discovery of potent inhibitors targeting S31N mutant strains is challenging but making progresses [84,85,86,87].

The viral ribonucleoprotein complex (vRNP) is composed of viral RNA, polymerase basic protein 2 (PB2), polymerase basic 1 (PB1), polymerase acidic (PA) proteins, and nucleoprotein (NP). Since the replication and translation of influenza viruses occur in the host nucleus and depend on the host RNA processing machinery, acquiring mutations in vRNP and the re-assortment of vRNP segments is critical for the adaption to a new host, especially when establishing a zoonotic transmission. The 1957 H2N2 Asian pandemic and the 1968 H3N2 Hong Kong pandemic were not only caused by the re-assortment of HA or NA, but also by the addition of a novel PB1 segment [88]. Mutations in PB2 protein, such as 627K and 701N, separately or in combination with NP mutations, have been shown to increase polymerase activity, thereby promoting avian H5N1 and H7N9 influenza virus replication, virulence, and transmission [89,90,91,92,93,94,95]. The PA subunit has also been related to viral pathogenicity and host adaption. For example, the re-assortment of avian polymerase with the PA subunit from a human 2009 H1N1 isolate or a 522S mutation in PA could overcome the species barriers [96]. Mutations in the PA protein have affected the virulence of avian H5N1 influenza virus [97,98]. Furthermore, a recent study reported that deletion mutations in PA resulted in strongly attenuated and temperature-sensitive viruses which could be safe candidates for future live attenuated influenza vaccines [99].

Influenza viral NS1 protein is a nonstructural protein which has multiple functions, including RNA binding, modulation of virus pathogenesis and host type I interferon inhibition. Identification of new mutations in NS1 protein is very important for understanding the pathogenicity of viruses and host switching [100,101]. Previous studies have demonstrated that several mutations in the NS gene were related to increased pathogenicity [102,103,104]. Mutations of F103L and M106I in human H5N1 isolates increased the viral replication and virulence [105]. Nevertheless, several mutations in the NS1 protein of seasonal influenza H3N2 virus have been shown to induce higher antiviral interferon responses and reduced viral virulence, and these attenuating mutations provided a new way to make live flu vaccines [101,106]. Meanwhile, the recombinant influenza viruses which contained the NS1-truncated mutants starting from the N-terminal could be used as live attenuated vaccines that provide protection against different strains of influenza A and B viruses [107,108,109].

## 3. Strategy for Influenza Vaccine

### 3.1. Current Influenza Vaccine

Current seasonal trivalent influenza vaccine (TIV) is composed of inactivated influenza vaccines or live attenuated influenza viruses from two influenza A strains (H3N2 and H1N1) and an influenza B strain which is recommended by WHO annually. Besides the TIV, quadrivalent influenza vaccines and pandemic influenza vaccines have been utilized in the current market as well. The recommended vaccine compositions are based on the comprehensive consideration of vaccine antigenicity, immunogenicity, production ability, and predictions based on influenza surveillance data [110].

### 3.2. New Strategy of Influenza Vaccine

With an advanced understanding of influenza virus genetic structure and immunogenicity, new strategies based on the conserved domain of different influenza virus proteins are being investigated, such as the HA stalk, M2e ectodomain, HA head COBRAs (Computationally optimized broadly reactive antigen), and NA-based influenza vaccines. These new strategies mainly aim to induce strong humoral immune responses. In addition, cellular immune responses, especially memory immune response, could play very important roles in the protection against infections by influenza. Some studies have demonstrated that the formulation containing peptides from influenza NP, PA, or M1 combined with different adjuvants could induce increased cytotoxic T lymphocyte (CTL) response, reduce lung viral titer, and provide protection against influenza virus infection [111,112,113,114,115]. A newly designed influenza vaccine, “Multimeric-001”, is based on the conserved epitopes from influenza HA, M1, and NP protein and was able to induce strong humoral and cellular immune responses [116]. The combination usage of Multimeric-001 with different adjuvants or conventional influenza vaccines by diverse immune stratagems, such as heterologous prime-boost or sequential immunization method, provides multiple ways to achieve universal influenza vaccines. Similarly, other vaccines based on the engineering of conserved peptides from influenza virus proteins, such as FP-01.1 and M2e multiple antigen peptides, will be strong universal influenza vaccine candidates [117,118].

Advantages of skin vaccination have been well-recognized in recent decades. Skin harbors abundant blood vessels, lymphatic vessels, as well as many different immune cells which are crucial for the regulators of both innate and adaptive immunity [119,120]. Skin vaccination has the potential to induce a strong innate immune response and a lasting cellular immune response. The biodegradable dissolving microneedle patch (MNP) is a novel technology for skin vaccination, and the application of vaccine encapsulated MNPs has been proven to induce increased immune responses and greater protective efficacy than the traditional intramuscular injection [121,122,123]. In our current study, we have demonstrated that a boosting skin vaccination with M2e-based vaccine encapsulated MNP could broaden immune responses and provide extra protection to the recipients who have received the traditional influenza vaccines prime [124]. Moreover, vaccine encapsulated MNPs are non-toxic, painless, stable, self-administrated, and easily produced. These features of MNPs, their ease of application, their shelf stability, and the rapid adaptability of the manufacturing process make MNPs a promising alternative to current soluble vaccines.

There are two major challenges for the development of a universal influenza vaccine. As we have mentioned above, the constant evolution of the influenza virus is one major reason that can render influenza vaccines ineffective when antigenic drift or shift occurs within circulating strain populations. On the other hand, the elderly, pregnant women, and children are the most vulnerable populations with decreased immune responses and protection efficacy after vaccination when facing influenza viral infection. Thus, the improved universal influenza vaccine is needed to confer protection for different human populations. A universal influenza vaccine based on the conserved regions of the virus could provide cross-protection against different strains, induce long-term immunity, and reduce vaccine production time. The delivery of the above novel vaccine formulations combined with the seasonal vaccine by novel immune strategies is a promising proposal for universal influenza vaccines.

## 4. Conclusions

Influenza vaccines are effective strategies for preventing infection. However, the selection of appropriate vaccine strains that match the probable circulating influenza strain in the impending flu season has many challenges. It mostly depends on worldwide influenza surveillance data, but the reformulated vaccines may fail to provide expected protection owing to viral antigenic drift, especially when a new pandemic virus emerges. In the future, a better understanding of the relationship among genomic sequences, structure, and function of each influenza virus protein is required for a better understanding of viral evolution, transmission, host switching, and pandemic formation. Deep understanding of all aspects of influenza antigenic proteins will provide guidance for the prediction of potential seasonal or pandemic influenza strains and the production of specific or universal influenza vaccines.

## Figures and Tables

**Table 1:** Summary of critical mutation sites for the function of influenza viral proteins.

Influenza Viral Protein	Functions	Mutation Sites	Conservation Sites	Applications in Vaccines
Hemagglutinin (HA)	Receptor binding, Membrane fusion	Receptor binding site, Glycosylation site, Proteolytic cleavage site	Stalk domain	HA stalk based vaccines, HA head COBRAs * Live attenuated vaccine strains
Neuraminidase (NA)	Virus releasing, Prevention aggregation, Penetration through mucus layer	Deletion in stalk domain, Glycosylation in stalk domain, Surface loops surrounding the enzyme active site	Enzymatic active site	Induction of NA immunity, Conserved epitopes in enzymatic site
Matrix 2 (M2)	Ion channel protein, Viral uncoating, Maintaining HA configuration, Virion budding and scission	Amantadine-resistant mutations V27A, S31N, and L26F	Ectodomain, An amphipathic helix in cytoplasmic tail	M2e ectodomain based vaccines
Viral ribonucleoprotein complex (vRNP)	NP-single strand RNA binding protein	NP-309K, 50G Temperature sensitive mutations		Live attenuated vaccine strains Conserved peptides in NP, PA and PB
	PB1-RNA dependent RNA polymerase	PB1-105S		
	PB2-binding host mRNA caps	PB2-627K, 701N, 591K		
	PA-essential for polymerase function	PA-552S, 224P, 383D		
Nonstructural 1 protein (NS1)	RNA binding, Type I interferon antagonism, Enhancing viral RNA translation, Inhibition of host mRNA processing	NS1-S42P, D92E, V149A NS1-103L, 106I		Live attenuated vaccine strains

* COBRA: Computationally optimized broadly reactive antigen.
